# Life cycle inventories and life cycle impact assessment datasets of a classic processing route for the manufacture of infant milk formula powder and of a low-heat alternative route involving the substitution of pasteurization by microfiltration

**DOI:** 10.1016/j.dib.2025.111892

**Published:** 2025-07-17

**Authors:** Michèle Gaillard, Emma Saint-Preux, Amélie Deglaire, Emeline Goussé, Gaëlle Tanguy, Nadine Leconte, Geneviève Gésan-Guiziou, Fanny Guyomarc’h, Juliane Floury

**Affiliations:** INRAE, Institut Agro, UMR STLO, 35042 Rennes, France

**Keywords:** Environmental assessment, LCA, LCI, Dairy processes, Baby food, Ecodesign

## Abstract

Infant milk formulas are industrial substitutes to human milk, used when breastfeeding is neither possible nor desirable. They are manufactured using successive additions of dry ingredients and oils to skim bovine milk, blending, pasteurization, homogenization, concentration by vacuum evaporation and spray-drying. However, successive heat loads are detrimental to the proteins’ nutritional quality of the final product. An alternative processing route has been proposed that replaces the first pasteurization step at 85 °C by a microfiltration step at 50 °C, i.e. below the heat-denaturation temperature of milk proteins. This data paper provides extensive description of the two processing routes at a semi-industrial scale, using measurements, calculations of the material and energy balances, expert say information, or proxies collected at UMR STLO in the period 2020–2024 and organized into life cycle inventories of all the data required for modelling. This dataset further provides the potential environmental impacts of 1 kg of the classic infant milk powder, as well as of 1 kg of infant milk powder produced using low-heat processing route as an alternative product. The Life Cycle Assessment (LCA) standard method was used, with the CML-IA, ReCiPe 2016, Impact World+, LC-Impact or EF3.0 characterization methods and a dry matter allocation rule for the skimming operation. The dataset further provides variations of the inventories and environmental impact assessments to evaluate the sensitivity of the results to the formula’s dry matter content prior to spray-drying or to the liquid *versus* dry state of the whey ingredient involved in the formula. The dataset participates in providing LCI of industrial food processes to the public. It will also be helpful for eco-designing classic or innovative processing routes for the manufacture of environmentally sustainable infant milk powders.

Specifications Table

**Table:** 

Subject	Engineering & Materials science
Specific subject area	Environmental assessment of food processing routes
Type of data	Table Raw, Analysed.
Data collection	This datapaper supports the research paper by Gaillard et al. [1]. The classic and alternative routes were described in Le Roux et al. [2] and Yu et al. [3]. They both resulted from experimental design and measurements made at the STLO Dairy Platform and the BIONOV SA semi-industrial facility in the 2018–2021 timeframe. Additional consumption data were: • Ingredients from mass and dry matter balances, • water (measures of time and mass), • energy (wattmeter or calculation from power, time and temperature), • cleaning products, using expert say and technical data, • metals, ceramic and rubber, from time measurement and technical data on materials and total lifetime. Data for liquid lactose and liquid whey were taken in Guyomarc’h et al. [4]. Background data were taken in the Ecoinvent 3.10 [5] and Agribalyse 3.1.1 databases [6]. The EF 3.0 characterization method was used in the Simapro Analyst software 9.5.0.1. Comparative analysis was conducted using other widespread characterization methods.
Data source location	Foreground data stored by UMR 1253 Science et Technologie du Lait et de l’Œuf (STLO) • Institution: INRAE-Institut Agro • City/Town/Region: Rennes • Country: France • Latitude : 48° 6′ 48.51″N and longitude : 1° 40′ 32.55″W Background data from databases (selected proxies: France, if not: Europe and if not: Global)
	Agribalyse 3.1.1 • Institution: ADEME • City/Town/Region: Nantes • Country: France • https://agribalyse.ademe.fr
	Ecoinvent 3.10 • Institution: Ecoinvent • City/Town/Region: Zurich • Country: Switzerland • https://ecoinvent.org
Data accessibility	Repository name: Recherche DataGouv / INRAE Dataverse / UMR-STLO Dataverse Data identification number: https://doi.org/10.57745/TAKOSX [7] Direct URL to data: https://entrepot.recherche.data.gouv.fr/privateurl.xhtml?token=1f4a7e0f–6345–427f-b0c0-eecef47299fc Instructions for accessing these data: Please fill in the dataset guestbook
Related research article	M. Gaillard, E. Saint-Preux, A. Deglaire, E. Goussé, G. Tanguy, N. Leconte, G. Gésan-Guiziou, F. Guyomarc’h, J. Floury, Life cycle assessment of a semi-industrial infant milk formula powder and of a low-heat alternative process involving membrane filtration, Cleaner Environmental Systems (2025) 100,278. https://doi.org/10.1016/j.cesys.2025.100278 [1].

## 1 Value of the Data

• This dataset provides a complete LCI data of two production routes of infant formula powders on a semi-industrial scale, a realistic scale for 100-kg to 1-ton batches.
• Only two precedent papers by and Karlsson et al. [8] and Andresen et al. [9] released LCI and LCA data for infant milk powder production. In these papers, only the classic route is considered and the LCI data are aggregated (e.g. “processing” aggregated all operations in one vector). Noteworthy, the present dataset also releases LCI and LCA data for a low-heat alternative route.
• Furthermore, the present LCI and LCA dataset examine the system to the detail of individual unit operations, even splitting each unit operation into three phases: the transient, stationary and cleaning phases, as to allow scalable calculations e.g. for industrially realistic processes where the stationary phase is typically hours long.
• LCA practitioners may use the data as proxies for other dairy-related life-cycle inventories.
• LCA practitioners may use the data for the eco-design of the production of infant formula milk powders.
• The data provides supplementary material to support the analyses and interpretations presented in Gaillard et al. [1].

## 2 Background

Infant milk powders are used to complete or replace human milk when required or chosen. Biomimicry is therefore a major challenge to ensure all nutritional and biological functions of human milk. In this perspective, replacing heat-treatment at 85 °C of the substitute bovine skim milk by microfiltration at 50 °C make it possible to sanitize the milk without significant alteration of the nutritional quality of the proteins [3]. Sourcing low-denatured whey proteins is also an issue, calling for specific preparation instead of cheese whey valorization. However, changes in the processing routes are expected to induce variation in the environmental footprint of the final infant milk powder. It was therefore decided to conduct comparative LCAs of a classic or an alternative route, the latter using microfiltration and low-heat liquid whey. The objective of this dataset is to release detailed semi-industrial data, at the level of single unit operations, to establish life cycle inventories relevant for the identification of environmental hotspots in the system, for testing processing parameters and for the eco-design of infant formula milk powder. These data were used in a research article [1] where the two production routes are analysed and compared.

## 3 Data Description

The dataset contains all the data related to the LCIs, LCAs and Monte Carlo simulations of semi-industrial production of infant formula milk powders using either a classic or an alternative processing route. The data are available in the following files:

1. LCI_System_and_Unit_operation_Numbers_and_Abbreviation: this is a single-sheet table file showing the operational cascade (also shown in Fig. 1) as well as the number, abbreviation and full designation of each operation as used in files 7 and 9.

**Fig. 1:**
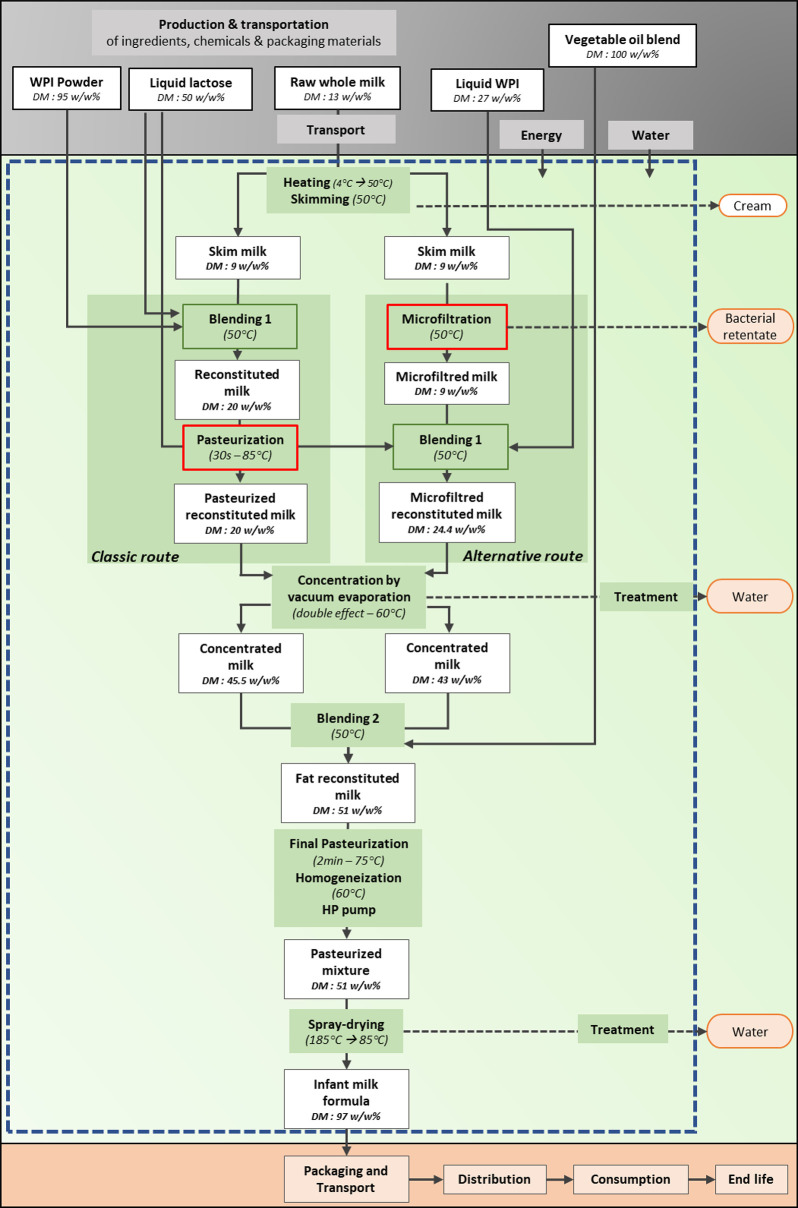
Illustration of the global system under study for infant formula production (FU: 1 kg), with specific difference in the 2-production routes in the green boxes. The production phase of the life cycle is depicted in the green box, while raw materials and inputs are enclosed in a grey box. The orange box present phases of the life cycle and products excluded from the study. Some operational parameters such as dry matter content (DM, in w/w %), temperature (in °C) and pasteurization time (in s), are also indicated.
2. LCI_Classic_route_1kg of infant formula: this is a single-sheet table file showing the Life Cycle Inventory of the production of 1 kg of infant formula following a classic production route, obtained from [2] and completed by measurements and calculations as described in the text. This file contains raw materials data, energy and water consumptions and mass of equipment for each unit operation.
3. LCI_Alternative_route_1kg of infant formula: this is a single-sheet table file showing the Life Cycle Inventory of the production of 1 kg of infant formula following an alternative, low-heat production route, obtained from [3] and completed by a liquid whey proxy [4] and by measurements and calculations as described in the text. This file contains raw materials data, energy and water consumptions and mass of equipment for each unit operation.
4. LCI_Additional_WPI_drying_step_for_the_Classic_route: this is a single-sheet table file showing the extra processing consumptions needed to spray-dry liquid whey protein isolate (WPI) to be used for the classic production route. Thus, both the classic and alternative production routes involve the same whey resource, avoiding discrepancies when using database proxies (e.g. whey protein for feed, with little precision on the industrial origin).
5. LCI_ Dry Matter adjusted_Classic_route_1kg of infant formula_EF3.0 method: this table provides data calculated for the first sensitivity analyses. Notably, the dry matter content of blend 1 was adjusted to 24 % w/w in the original classic route, by adjusting the amount of water required to solubilize powder WPI. Thus, the environmental impacts of concentration by vacuum evaporation were equalized in both the classic and alternative routes, to help better comparing the first pasteurization and microfiltration. The data used for the original classic route has therefore been modified during this sensitivity analysis, for unit operations: blending n°1, pasteurization and concentration by vacuum evaporation. Their duration has been reduced and consequently energy, metals and water consumption have also been reduced.
6. LCI_ Dry Matter and WPI adjusted_Classic_route_1kg of infant formula_EF3.0 method: this table provides data calculated for the second sensitivity analyses. Notably, the impacts of involving WPI in the liquid versus powder form were evaluated in the classic route. In the initial classic route, powder WPI (95 % w/w dry matter) is solubilized into water until the final dry matter content of the WPI is 4.9 % w/w. To optimize this, powder WPI is replaced by liquid WPI (27 % w/w dry matter) in the classic route, without any further dilution to 4.9 %. The sensitivity test compared the original classic route (using WPI powder solubilized at 4.9 % w/w dry matter) with a “DM and WPI adjusted” classic route (using liquid WPI at 27 % w/w dry matter). The final dry matter contents of blend 1 was 20 % or 26.7 %, respectively, at the inlet of concentration by vacuum evaporation unit. The data used for the original classic route has therefore been modified during this sensitivity analysis, for unit operations: blending n°1, pasteurization and concentration by vacuum evaporation. Their duration has been reduced and consequently energy, metals and water consumption have also been reduced.
7. LCI_Databases_and_processus_for_the_Classic and Alternative routes_1kg of infant formula: this is a single-sheet table file providing all the processes and their origin databases (EcoInvent 3.10 and Agribalyse 3.1.1).
8. LCI_Quality_of_data_by_Pedigree_evaluation_matrix: this is a single-sheet table file providing the result of self-evaluation of the uncertainty of each data type using the Pedigree matrix. Distributions were assumed to be log-normal.
9. LCA_Classic_route_1kg of infant formula_EF3.0 method: this is a single-sheet table file showing the raw results of the environmental impact assessments for the production of 1 kg of infant formula following a classic production route, calculated with the EF 3.0 method in the SimaPro Analyst 9.5.0.1 software. Details for each unit operation and the 3-processing phases (transient, steady and cleaning phases) are also available.
10. LCA_Monte-Carlo simulation_Classic route_EF3.0 method: this is a single-sheet table file showing the raw results of the single Monte-Carlo simulation for the production of 1 kg of infant formula, following a classic production route. This simulation provided statistical data with 95 % confidence: mean, median, standard deviation, coefficient of variation, and the standard error of the mean.
11. LCA_Alternative_route_1kg of infant formula_EF3.0 method: this is a single-sheet table file showing the raw results of the environmental impact assessments for the production of 1 kg of infant formula following the alternative production route, calculated with the EF 3.0 method in the SimaPro Analyst 9.5.0.1 software. Details for each unit operation and the 3-processing phases (transient, steady and cleaning phases) are also available.
12. LCA_Monte-Carlo simulation_Alternative route_EF3.0 method: this is a single-sheet table file showing the raw results of the single Monte-Carlo simulation for the production of 1 kg of infant formula, following the alternative production route. This simulation provided statistical data with 95 % confidence: mean, median, standard deviation, coefficient of variation, and the standard error of the mean.
13. LCA_comparative Monte-Carlo_Simulation_Classic and Alternative route_EF3.0 method: this is a single-sheet table file showing the raw results of the comparative Monte-Carlo simulation for the production of 1 kg of infant formula, following a classic or the alternative production route. This simulation provided statistical data with 95 % confidence: mean, median, standard deviation, coefficient of variation, and the standard error of the mean.
14. LCA_Dry Matter adjusted_Classic_route_1kg of infant formula_EF3.0 method
15. LCA_Monte-Carlo simulation_Dry Matter adjusted_Classic route_EF3.0 method
16. LCA_Dry Matter and WPI adjusted_Classic_route_1kg of infant formula_EF3.0 method: this is a single-sheet table file showing the result of a sensitivity test by which the WPI powder involved in the classic route in the form of a 4.9 % dry matter solution is replaced by liquid WPI at 27 % dry matter. In this test, the resultant effect of avoiding both the environmental impacts of WPI drying and WPI dilution is calculated.
17. LCA_Monte-Carlo simulation_Dry Matter and WPI adjusted_Classic route_EF3.0 method : this is a single-sheet table file showing the raw results of the single Monte-Carlo simulation for the production of 1 kg of infant formula, following a Dry Matter and WPI adjusted classic production route. This simulation provided statistical data with 95 % confidence: mean, median, standard deviation, coefficient of variation, and the standard error of the mean.
18. LCA_Classic and Alternative_route_1kg of infant formula_ReCiPe 2016: this is a single-sheet table file showing the raw results of the environmental impact assessments for the production of 1 kg of infant formula following a classic and an alternative production route, calculated with the ReCiPe 2016 method in SimaPro.
19. LCA_Classic and Alternative_route_1kg of infant formula_CML-IA: this is a single-sheet table file showing the raw results of the environmental impact assessments for the production of 1 kg of infant formula following a classic and an alternative production route, calculated with the CML-IA method in SimaPro.
20. LCA_Classic and Alternative_route_1kg of infant formula_Impact World+: this is a single-sheet table file showing the raw results of the environmental impact assessments for the production of 1 kg of infant formula following a classic and an alternative production route, calculated with the Impact World+ method in SimaPro.
21. LCA_Classic and Alternative_route_1kg of infant formula_LC-IMPACT: this is a single-sheet table file showing the raw results of the environmental impact assessments for the production of 1 kg of infant formula following a classic and an alternative production route, calculated with the LC-IMPACT method in SimaPro.
22. LCA_Robustness_of_LCA_results_across_characterization_methods: this is a single-sheet table file showing a comparison of calculated difference (in %) between the environmental impacts of 1 kg of infant milk formula depending on the production route (classic vs alternative). The differences are calculated for each characterization method considered in order to test the relevance of the EF 3.0 method.

## 4 Experimental Design, Materials and Methods

This work followed the International Standard Organization (ISO) 14,040 recommendations for applying the life cycle assessment (LCA) method [10].

### 4.1 Goal and scope

The goal of this study was to assess and compare the potential environmental impacts of a semi-industrial production of infant milk formula powder using attributional LCA, depending on whether the skim dairy base was sanitized by heat-treatment (classic route) or by microfiltration (alternative route). As largest contributions to the potential environmental impacts were notably generated by water removal rather than sanitation itself, the study further compared variations of the classic routes as sensitivity tests. Namely, processing parameters like the dry matter content prior to concentration by vacuum evaporation and/or the physical state of the whey ingredient were varied in the classic route. The scope is that of the French dairy industry with classical technologies and energy consumption mix in the 2010s period. The geographical scope is France, where electricity is mainly produced by nuclear power with low emissions of greenhouse gases. All raw materials are produced in France.

### 4.2 System boundaries

The system boundaries are farm-to-gate, from raw milk production at the farm to infant formula at the exit gate of the factory (Fig. 1). It includes milk production and transport, skimming, blending with other dairy ingredients (blend 1), sanitization of blend 1, concentration by vacuum evaporation, addition of oils to yield blend 2, final pasteurization, homogenization and spray-drying. The cream co-produced at the skimming stage exits the system using a dry matter allocation rule. The retentate co-produced at the microfiltration stage exits the system as a waste as it is meant to concentrate and remove the contaminating bacteria. The main differences between the classic and the alternative routes are presented in Fig. 1 as diverging flows between skimming and concentration by vacuum evaporation. Packaging and transport of the manufactured products, distribution, consumption and product’s wastage or end-of-life were excluded. Maltodextrin and micronutrients like vitamins or trace elements, usually incorporated in blend 2, were omitted due to lack of information.

### 4.3 Functional unit

The functional unit was 1 kg of unpackaged infant formula milk powder produced at a semi-industrial scale.

### 4.4 General description of the processing routes to produce the functional unit

Fresh raw whole milk was produced by conventional dairy farms in Brittany, collected by the SODIAAL dairy plant in Montauban-de-Bretagne, France, where it was purchased as a cooled raw blend. It was transported over 30 km to the semi-industrial dairy processing unit, BIONOV S.A. (Rennes, France). The milk was heated from 4 °C to 50 °C and skimmed using a MSE 25–01–177 Westfalia separator (GEA, Chateau-Thierry, France). The resulting skimmed milk was kept for formulation while the cream exited the system as a co-product.

In the classic route, dried whey protein isolate (WPI) was solubilized, then subsequently blended with skimmed milk and liquid lactose, resulting in a 20 % w/w dry matter content reconstituted milk base, which was pasteurized at 85 °C for 30 s [2].

In the alternative route, skimmed milk was first subjected to microfiltration up to a volume concentration factor of 20 at a temperature of 50 °C, using a 0.8 µm ceramic membrane (Pall Corporation, Bazet, France). The permeate (i.e. sanitized skim milk) was retained, while the bacterial retentate exited the system as a waste. Liquid WPI and lactose were subsequently incorporated, leading to a 24 % w/w dry matter content.

Each route therefore yielded a sanitized milk base (blend 1) that was then concentrated by a two-effect evaporator (GEA Process Engineering, St Quentin-en-Yvelines, France), reaching approximately 45 % and 43 % w/w dry matter for the classic and alternative routes, respectively. Then, a blend of vegetable oils was added to each formula to ensure a balanced fat supply, and this blend 2 at 51 % w/w dry matter was homogenized at 60 °C using a two-stage homogenizer at 14 and 4 MPa (VMI Raynerie Multimix 400 62,601, Montaigu, France). The homogenized blend 2 then underwent final pasteurization at 75 °C for 2 min (Eurotransfert tubular exchanger, 2 sections of 2 tubes, Toulouse, France) and spray-drying, using a semi-industrial-scale 2-stage spray-dryer equipped with a fluidized bed (Niro Atomizer, GEA-PE, Saint Quentin en Yvelines, France) to achieve a final infant formula powder with a dry matter content of 97 % w/w. The theoretical maximum evaporation capacity was approximately equal to 90 kg·h⁻¹ of water, and the air temperatures at the dryer inlet and outlet were set at 165 °C and 85 °C, respectively.

A single production batch lasted half a day and yielded approximately 62.5 kg of infant formula powder meeting the required quality standards. The available data in this paper include the transient phase (up to full production regime), the steady phase of production and the cleaning phase. The reference flows were converted to produce 1 kg of infant formula powder. Mass and composition of the raw materials are presented in Table 1.

**Table 1:** Mass and composition of raw materials for the classic and the alternative routes for the production of 1 kg of infant formula.

Raw materials	Classic route		Alternative route	
	Mass (kg)	Dry matter content (w/w %)	Mass (kg)	Dry matter content (w/w %)
Raw milk	2.9	13 %	3.4	13 %
Whey Protein Isolate (WPI)	9.5 × 10^−2^ (powder)	95 %	0.35 (liquid)	27 %
Liquid lactose	1.4	50 %	1.5	50 %
Oil mix	0.34	99.9 %	0.4	99.9 %
Water	1.74	/	/	/
Maltodextrin	0.15	95 %	/	/
Minerals	/	/	0.03	100 %

Maltodextrins and minerals, typically present in industrial infant formulas, were omitted in [2] and in this study. Due to their by-product origin (maltodextrin) and low incorporation rate (minerals), we assumed that their potential environmental impact was negligible. However, they were taken into account in the masse balance sheets (section 5.1.)

### 4.5 Life cycle inventory

#### 4.5.1 Relevant literature

Data for the classic route were taken in [2] while data for the alternative route were from [3]. Both studies reported experiments that were conducted on the same facilities: STLO platform and BIONOV SA.

#### 4.5.2 Raw materials

Some modifications have been made to the physical properties of raw materials to better address the goals of the LCA. For the classic route, the skimmed cow milk powder used in [2] was replaced by whole raw milk to better reflect industrial practices. Lactose was introduced as a liquid solution instead of a powder in order to align the two production routes and to mainly focus on the environmental impacts of manufacturing processes.

The alternative route was designed to minimize thermal processing. It used exclusively liquid raw materials, thereby omitting concentration by vacuum evaporation and drying steps of the input ingredients WPI and lactose. Data for the production of liquid lactose and WPI were taken from [4] and [11], also obtained at UMR STLO. When necessary, they were adapted to convert liquid WPI into powder WPI, using [12] for energy data and [11] for materials and process water data of concentration by vacuum evaporation and spray-drying.

Quantities needed to produce 1 kg of IF were calculated from mass balances performed on each unit operation using data from [2] for the classic route and from [3] for the alternative route. These mass balances were calculated based on the total dry matter content and mass of each raw materials in all inputs and outputs using (1), (2):

• Overall mass balance:
  (1) $$\sum m_{\text{inputs}}=\sum m_{\text{outputs}}$$
• Specific mass balance:
  (2) $$\sum \left(m_{\text{input}}\times DM_{\text{input}}\right)=\sum \left(m_{\text{output}}\times DM_{\text{output}}\right)$$

with DM: dry matter

As skim milk is 70 % of the total dry matter of the raw milk, a 70 % dry matter allocation factor has been applied on raw milk to exclude the environmental impacts of the cream co-produced during the skimming process [4].

All other inventory data for raw materials were taken in the Agribalyse 3.1.1 and Ecoinvent v3.10 databases [5,6].

#### 4.5.3 Transport

Only the transportation of raw milk to the UMR STLO was considered in the LCI, for which a non-refrigerated van completed a 60-km return journey. The transports of WPI, lactose and oil blend were excluded from the system.

#### 4.5.4 Processing

BIONOV SA is fully powered by electricity. It operates at a semi-industrial scale, where milk processing operations are conducted on daytime hours and not with 3 × 8 h continuous flow. Therefore, the initialization time of the equipment (so-called transient phase) is not negligible in relation to the total duration of use of equipment at semi-industrial scale. In order to fully understand the origin of environmental impacts for each unit operation of the processing phase, the life cycle inventory was divided into 3 distinct phases: the transient phase, steady phase and cleaning phase.

##### 4.5.4.1 Transient phase and steady phase

For each of these phases and for each unit operation: the duration of the phase, the energy, water and machine requirements, machine materials and total lifetime were taken into account in the LCI, with in addition the raw materials for the steady phase.

These data have been collected from expert reports generated through on-site experiments at the Dairy Platform and BIONOV. Based on the machines used and available data, energy balances were used to calculate the energy consumption for each operation [13].

To estimate the energy consumption, Q, of:

• The pumps and motors in the equipment, the following equations were employed:
  (3) $$Q(\mathrm{kWh})=\left(\left(I\times U\times \sqrt{3}\times \cos\Phi \right)/1000\right)\times t$$
  (4) Q(kWh)=P×t
• The pasteurization and spray-drying equipment:
  (5) $$Q_{2}\left(\mathrm{kWh}\right)=\left(Q_{1}\times t_{2}\right)/t_{1}$$

With Q_1_ the energy (in kWh) consumed by the operation for a certain time (t_1_ in hours) and t_2_ the operating time of the equipment in production (in h).

• The heat exchange to a considered mass of product:
  (6) $$Q\left(\mathrm{kWh}\right)=\left(m\times \mathrm{Cp}\times \left(\theta_{\mathrm{final}}-\theta_{\mathrm{initial}}\right)\right)/3600$$

With m the mass of product to be heated (in kg), Cp the heat capacity of the product (in kJ.kg^−1^.K^−1^), and θ_initial_ or θ_final_ the initial or final temperature of the product, respectively (in °C).

For the temperature-holding steps in the mixing operations, the following equation, derived from Fourier’s law for heat conduction, was used:

(7) $$Q\left(\mathrm{kWh}\right)=A\times \left(k/z\right)\times \left(T_{\mathrm{inside}}-T_{\mathrm{outside}}\right)\times t$$

With A the reactor area (in m^2^ – [14]), k the thermal conductivity of the insulation material (in W.*m*^−1^.K^−1^), z the insulation thickness (in m), T_inside_ the temperature inside the vessel (in °C), T_outside_ the temperature outside the vessel (in °C) and t the mixing time (in h).

##### 4.5.4.2 Cleaning phase

The duration of the cleaning phase, the energy, water and machine requirements were taken into account in the LCI, for each unit operation.

At BIONOV, some pieces of equipment do not have a cleaning-in-place station (CIP): the first heat exchanger (for heating raw milk), the cream separator, tanks for blending and spray-drying tower. They are manually cleaned after a production day, the product in the equipment is first removed by a water push, followed by a rinse, then soda wash (20 g.L^−1^), second rinse, acid wash and final rinse. Chemicals were single used and recovered in wastewater. Water consumption and discharges, including detergent solutions, were quantified by on-site measurements, including the water used for cooling and cleaning.

##### 4.5.4.3 Additional hypotheses

In addition, the following hypotheses were put forward:

• Energy consumption losses in primary heat-exchange loops to heat transfer fluids was neglected;
• Energy consumption during maintenance (excluding cleaning) was neglected;
• For cleaning operations, the energy consumption required to maintain water and detergent at constant temperatures was considered negligible compared to heating;
• The equipment was assumed to be used at its maximum power;
• The use of the equipment, and hence the consumption of materials and metals, was calculated *prorata temporis* of their total lifetime. Materials, weight, consumables and lifetimes of the equipment and consumables were found in technical data or expert say.

### 4.6 Life cycle impact assessment

Evaluation of the potential environmental impacts of infant milk formula by LCA was performed using the Environmental Footprint 3.0 characterization method (EF 3.0 adapted version 1.03) using the SimaPro Analyst software release 9.5.0.1 (PRé Sustainability, Amersfoort, The Netherlands) loaded with the Agribalyse 3.1.1 and EcoInvent 3.10 databases. These tools were all provided by the INRAE-CIRAD Multi-Criteria Assessment of Sustainability platform (MEANS). The results were compared to those obtained with the following characterization methods to test the robustness of the evaluation: ReCiPe 2016 Endpoint (H) v1.07, CML-IA baseline v3.08, Impact World+ Midpoint v1.02 and LC-Impact 100y v1.01.

### 4.7 Sensitivity analysis

Two sensitivity analyses were conducted in this work. In the first analysis, the dry matter content of blend 1 was adjusted to 24 % w/w in the classic route, i.e. equal to that of the alternative route, so that there was no difference between the two routes with respect to the dry matter content of blend 1 before concentration by vacuum evaporation. This test was therefore a “dry matter adjusted” classic route. To do this, only 0.66 kg of water were input in blend 1 of the classic route, instead of 1.74 (Table 1). Overall, the volume of blend 1 is reduced. Therefore, durations of the preparation of blend 1 and of the combined pasteurization + concentration by vacuum evaporation steps were assumed to be reduced, assuming a constant feed’s flow-rate and constant performance for each operation. Then, power and resources consumptions were recalculated as described above.

In the second analysis, the powder WPI used in the classic route was replaced by liquid WPI, i.e., the same ingredient was used as in the alternative route. In the initial classic route, powder WPI (95 % w/w dry matter) is solubilized into water until the final dry matter content of the WPI is 4.9 % w/w. Of course, significant amounts of water are therefore evaporated twice: first to produce the powder WPI, then to concentrate blend 1 where the 4.9 %-WPI is incorporated (Fig. 1). To optimize this, powder WPI is replaced by liquid WPI (27 % w/w dry matter) in the classic route, without any further dilution to 4.9 %. As a consequence, the sensitivity test compared the original classic route (using WPI powder solubilized at 4.9 % w/w dry matter) with a “DM and WPI adjusted” classic route (using liquid WPI at 27 % w/w dry matter). The final dry matter contents of blend 1 was 20 % or 26.7 %, respectively, at the inlet of concentration by vacuum evaporation unit. This test was therefore a “dry matter and WPI adjusted” classic route. Overall, the volume of blend 1 is reduced. Therefore, durations of the preparation of blend 1 and of the combined pasteurization + concentration by vacuum evaporation steps were assumed to be reduced, assuming a constant feed’s flow-rate and constant performance for each operation. Then, power and resources consumptions were recalculated as described above.

## Limitations

BIONOV SA is a semi-industrial facility and it is operated only on daytime hours. Therefore, equipment downtime significantly exceeds operational periods during a typical production day. A first limitation to this study is that resource consumptions related to ramping up the various unit operations and to cleaning are proportionally greater in the present study, reported to the functional unit, than in true dairy industrial processes, where facilities are often driven 2 × 8 or 3 × 8 h a day. This limitation has been corrected with the separate inventory of the transient, steady and cleaning phases; for users who would wish to adapt the present data set to industrial conditions. However, the fact that some pieces of equipment were not connected to a CIP facility (see 5.4.2) make some data difficult to scale-up.

Furthermore, BIONOV SA is fully powered by electricity, while industrial facilities use a significant share of natural gas, fuel and/or wood pellets to generate heat. Due to this difference in energy mix, the potential environmental impacts of BIONOV SA are greater in electricity-related impact categories, e.g. the emission of ionizing radiations (French electricity mix), and lower in impact categories related to the combustion of fossil resources, e.g. emissions of greenhouse gas, particulate matter, etc.

Except for the cream, to which some environmental impacts are attributed using dry matter allocation, and the WPI, which is issued of a background process where dry matter allocation was also applied, all food losses are regarded as wastes. In industrial conditions, losses would be reduced, and uncompressible losses would be valorized e.g. into feed or biogas production.

Finally, a limitation of the study lays in the accessible boundaries, as no downstream processes such as packaging and transportation were considered. The final use of the product was not considered either, whereas infant formula milk is a fragile product once reconstituted and is thrown away if the baby does not finish the bottle. At this stage of research, there is no evidence that expiry date and spoilage would differ between the classic and the alternative route, so consumption was not regarded as discriminant. However, farm-to-grave boundaries would have proportionally reduced the sanitization’s share of potential environmental impacts.

## Ethics Statement

The authors comply to the Data in Brief’s ethical requirements and confirm that the current work does not involve human subjects, animal experiments, or any data collected from social media platforms.

## CRediT authorship contribution statement

**Michèle Gaillard:** Conceptualization, Methodology, Investigation, Data curation, Writing – original draft. **Emma Saint-Preux:** Conceptualization, Methodology, Investigation, Data curation. **Amélie Deglaire:** Conceptualization, Investigation, Resources, Funding acquisition, Writing – review & editing. **Emeline Goussé:** Conceptualization, Investigation, Resources, Writing – review & editing. **Gaëlle Tanguy:** Conceptualization, Investigation, Resources, Writing – review & editing. **Nadine Leconte:** Conceptualization, Investigation, Resources, Writing – review & editing. **Geneviève Gésan-Guiziou:** Conceptualization, Investigation, Resources, Funding acquisition, Writing – review & editing. **Fanny Guyomarc’h:** Conceptualization, Methodology, Supervision, Data curation, Writing – review & editing. **Juliane Floury:** Conceptualization, Methodology, Investigation, Resources, Supervision, Data curation, Writing – review & editing.

## Data Availability

DataverseLCI and LCIA Datasets for Conventional and Microfiltered Processing of Infant Formula Powder (Original data).
